# The global power sector’s low-carbon transition may enhance sustainable development goal achievement

**DOI:** 10.1038/s41467-023-38987-4

**Published:** 2023-05-30

**Authors:** Kun Peng, Kuishuang Feng, Bin Chen, Yuli Shan, Ning Zhang, Peng Wang, Kai Fang, Yanchao Bai, Xiaowei Zou, Wendong Wei, Xinyi Geng, Yiyi Zhang, Jiashuo Li

**Affiliations:** 1 Institute of Blue and Green Development, Shandong University, Weihai, 264209 China; 2 Department of Geographical Sciences, University of Maryland, College Park, MD 20742 USA; 3 Fudan Tyndall Center, Department of Environmental Science and Engineering, Fudan University, Shanghai, 200438 China; 4 School of Geography, Earth and Environmental Sciences, University of Birmingham, Birmingham, B15 2TT UK; 5 Key Lab of Urban Environment and Health, Institute of Urban Environment, Chinese Academy of Sciences, Xiamen, 361021 China; 6 School of Public Affairs, Zhejiang University, Hangzhou, 310058 China; 7 College of Environmental Science and Engineering, Yangzhou University, Yangzhou, 225127 China; 8 School of Environmental Science and Engineering, Shanghai Jiao Tong University, Shanghai, 200030 China; 9 Economics and Management School, Wuhan University, Wuhan, 430070 China; 10 Guangxi Key Laboratory of Intelligent Control and Maintenance of Power Equipment, Guangxi University, Nanning, 530004 China

**Keywords:** Environmental impact, Sustainability, Climate change

## Abstract

The low-carbon power transition, which is key to combatting climate change, has far-reaching effects on achieving the Sustainable Development Goals (SDGs) in terms of issues such as resource use, environmental emissions, employment, and many more. Here, we assess the potential impacts of the power transition on progress toward achieving multiple SDGs (covering 18 targets across the 17 goals) across 49 economies under nine socioeconomic and climate scenarios. We find that the low-carbon power transition under the representative concentration pathway (RCP)2.6 scenarios could lead to an approximately 11% improvement in the global SDG index score from 54.70 in 2015 to 59.89-61.33 in 2100. However, the improvement would be significantly decreased to 4.42%-7.40% and 7.55%-8.93% under the RCP6.0 and RCP4.5 scenarios, respectively. The power transition could improve the overall SDG index in most developed economies under all scenarios while undermining their resource-related SDG scores. Power transition-induced changes in international trade would improve the SDG progress of developed economies but jeopardize that of developing economies, which usually serve as resource hubs for meeting the demand for low-carbon power transition in developed economies.

## Abstract (web summary)

The low-carbon power transition could enhance global sustainable development goal (SDG) progress, but hinder that of developing economies under fossil fuel-based scenarios. Meanwhile, SDG synergies and trade-offs exist within and between economies.

## Introduction

The current fossil fuel-dominated power sector accounts for nearly 40% of global annual energy-related CO_2_ emissions^1,2^. The low-carbon transition of the power sector is crucial to tackling climate change and ensuring the future supply of energy^3,4^. However, the impacts of power sector transition are beyond concerns for the climate. Power sector transition has far-reaching effects on achieving the Sustainable Development Goals (SDGs)^5^ in terms of issues such as resource use^6,7^, environmental emissions^4^, employment^8^, and many more^9,10^. However, the power transition may alleviate one problem while simultaneously exacerbating others. For instance, the closure of coal-fired power plants will reduce cooling water withdrawal (advancing the achievement of SDG 6: clean water and sanitation)^11,12^ but cause massive job losses in the coal power industry and its various ancillary, upstream, and downstream industries (hindering the achievement of SDG 8: decent work and economic growth)^13,14^. The expansion of low-carbon power such as wind power and solar energy as substitutes for fossil fuels can improve countries’ ability to address climate change (advancing the achievement of SDG 13: climate action)^15^ while increasing demand for critical materials (hindering the achievement of SDG 12: responsible consumption and production)^16,17^. Thus, there is a need to understand the impacts of the power transition on global SDG progress in terms of multiple aspects.

Previous studies have primarily focused on the impacts of specific national or regional power sector transitions on a single aspect of sustainable development, such as regional employment^18^, economic growth^19^, natural resource use^20,21^, and greenhouse gas and pollutant emissions^22^. Additional work is required to reflect the impacts of the power transition on SDG progress, particularly the environmental, social, and economic implications (trade-off or synergies) of a power sector transition and its impact on each region with respect to the multiple SDG goals. For instance, Wang et al.^23^ found that developing Asia’s short-term coal power plan has not yet included the impact on the regional sustainable use of water resources, which may exacerbate water shortages in Asia (hindering the achievement of SDG 6: clean water and sanitation) if no strategy is designed to reduce cooling water use. Second, a power transition in one region affects not only the achievement of local SDGs but also SDG progress in other regions via interregional trade. The expansion of renewable power or the reduction of fossil fuels in the electricity mix in one country might lead to changes in environmental pollution, resource consumption, and employment embodied in products and services from global supply chains, thus potentially influencing other regions’ achievement of the SDGs^24^. Some researchers have conducted initial investigations and found that European renewable energy directives may potentially harm forests in tropical countries, such as Indonesia and Brazil, through the wood trade (hindering the achievement of SDG 12: responsible consumption and production)^25^. Thus, exploring the role of international trade in regional SDG progress is vital for preventing a power transition at the expense of SDG achievement in other regions.

In this work, we make a methodological contribution by coupling the global change assessment model (GCAM)^26^, multiregional input-output (MRIO) analysis^27,28^ and the SDG approach^29^ to examine the direct and supply chain effects of power transitions throughout the world on achieving regional and global SDGs by 2100, including the net environmental and socioeconomic changes. Given its simplicity and transparency compared with other economic system accounting methods such as the computational general equilibrium model^30–32^, MRIO analysis is selected and used to capture the direct and supply chain effects of power sector activities on environmental and socioeconomic changes. The GCAM is used to simulate the power transition pathways in a scenario framework^33–35^, in line with the shared socioeconomic pathways (SSPs, e.g., SSP1, SSP2, and SSP5)^36,37^ and representative concentration pathways (RCPs, e.g., RCP2.6, RCP4.5, and RCP6.0)^38,39^. The selected pathways are meant not to cover all possible power sector futures but to demonstrate the effects of some power transition choices on SDG achievement in our analysis. Among many integrated assessment models (IAMs), the GCAM is selected for its transparency and credibility in multimodel, multiscale analysis, in which it is either soft- or hard-coupled to other models with different focuses and often greater resolution in power sectors^26,40^. Specifically, the GCAM and the MRIO model can achieve complementary advantages in scenario analysis^41^. The MRIO model can provide cross-regional impact analysis of the power sector in the base year, while the GCAM, as an IAM, can provide not only future changes in the size of the power sector but also future trends in some parameters that can be used in the MRIO model (such as CO_2_ emissions per unit of electricity generated in the power sector and blue water consumption). Finally, the changes in environmental and social-economic indicators driven by power transitions are translated into SDG progress using the United Nations SDG approach. Our findings demonstrate that the low-carbon transition of the global power sector could enhance overall SDG performance with enormous regional disparities in the individual targets of the SDGs.

## Results

### The integrated assessment framework design for the power transition

**Fig. 1:**
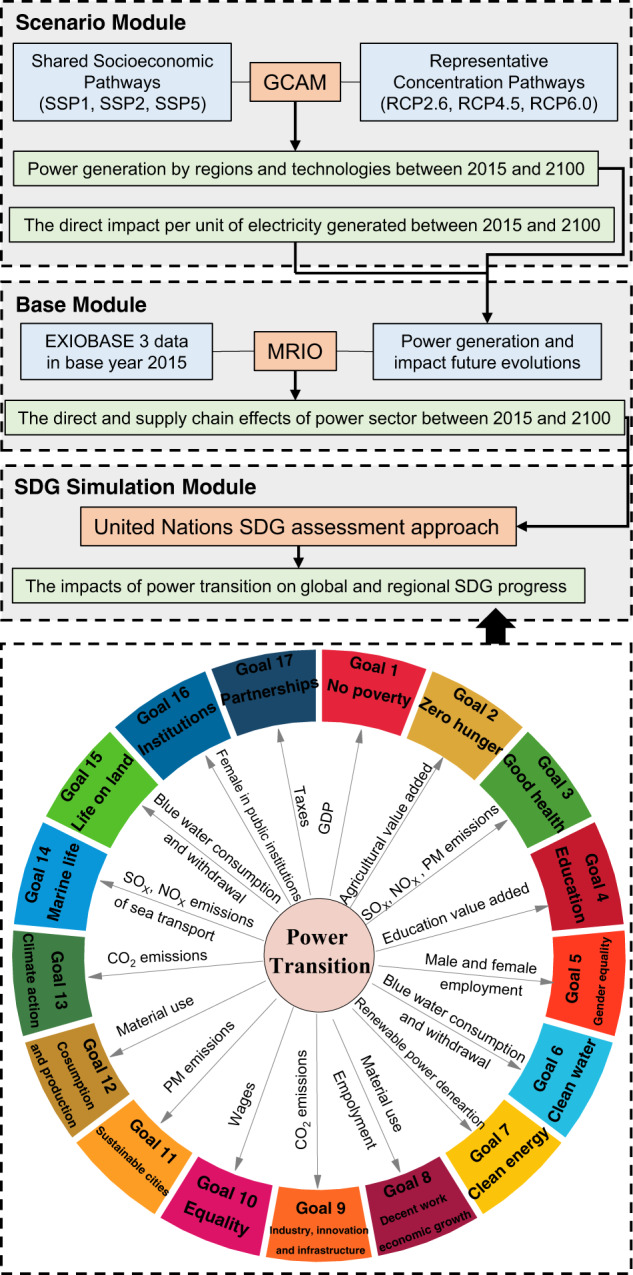
The integrated assessment framework of this study. An integrated assessment framework is designed to examine the direct and supply chain effects of power transitions throughout the world on the achievement of regional and global SDGs from 2015 to 2100. This framework includes three main modules: a base module, a scenario module and an SDG simulation module.

In this study, an integrated assessment framework is designed to examine the direct and supply chain effects of power transitions throughout the world on the achievement of regional and global SDGs from 2015 to 2100. This framework includes three main modules: a base module, a scenario module and an SDG simulation module (Fig. 1). The base module is used to describe the direct and supply chain effects of the global power sector, including the environmental and socioeconomic impacts; the scenario module is able to simulate the global power transition pathways and provide some of the parameters (for example, environmental impact per unit of electricity generated) for future evolutions for base module 1; and the SDG simulation module is designed to translate the changes in environmental and socioeconomic indicators into global SDG progress.

The base module is based on the EXIOBASE MRIO model^42^, which supports modeling the environmental and socioeconomic impacts of 10 main categories of power production subsectors, including coal power, gas power, nuclear power, hydroelectricity, wind power, petroleum and other oil derivatives power, biomass and waste power, solar photovoltaic (PV), solar thermal, and geothermal power. These environmental and socioeconomic indicators representing the SDGs include carbon dioxide (CO_2_) emissions, sulfur oxide (SO_X_) emissions, nitrogen oxide (NO_X_) emissions, particulate matter (e.g., PM_2.5_ and PM_10_) emissions, blue water withdrawal, blue water consumption, material use (fossil fuel, biomass, metal, and nonmetal), employment, value-added, taxes, and wages. MRIO analysis is selected for its transparency and credibility in analyzing the impacts of the power sector on the entire upstream and downstream value chain.

The scenario module based on the GCAM can simulate the global power transition pathways in a new scenario framework, and it has been frequently used to study energy^43^ and climate change issues^44,45^. The new scenario framework combines alternative pathways of socioeconomic development (i.e., SSPs) with pathways of future radiative forcing and their associated climate changes (i.e., RCPs). The SSPs are defined along two different dimensions: challenges to adaptation and challenges to climate mitigation. To take into account the general trend of globalization, SSPs with high (SSP5), medium (SSP2), and low levels (SSP1) climate mitigation challenges but with relatively low challenges to adaptation (SSP1 and SSP5) are considered. SSP2 is chosen as an intermediate path. In terms of the RCPs, three levels of scenarios (RCP6.0, RCP4.5, and RCP2.6 W m^–2^) are selected. RCP8.5 is excluded because it is intended to explore an unlikely high-risk future. As the available evidence does not yet indicate that the world has seriously committed to achieving the 1.5 °C goal^46^, RCP1.9 is not included. In general, the selected pathways (SSP5 + RCP6.0, SSP5 + RCP4.5, SSP5 + RCP2.6, SSP2 + RCP6.0, SSP2 + RCP4.5, SSP2 + RCP2.6, SSP1 + RCP6.0, SSP1 + RCP4.5 and SSP1 + RCP2.6.) are meant not to cover all possible power sector futures but to demonstrate the effects of some socioeconomic development and climate actions on the power transition in our analysis. Another important role of the scenario module is to provide future trends in some parameters in the MRIO model (such as CO_2_ emissions per unit of electricity generated in the power sector and bluewater consumption). The heterogeneity of parameter changes in different power sectors of different regions is comprehensively considered based on their previous characteristics and future possible conditions of socioeconomic and technological development in the selected SSP + RCP scenarios. The description of the parameter changes at the sector level is shown in Supplementary Data 1–7.

The SDG simulation module can translate the changes in environmental and socioeconomic indicators driven by the power transition into global SDG progress. After the results are output in the base module and scenario module, the SDG simulation module uses the SDG approach to transform and standardize the data. The role of data transformation is to transform the direct impact into an impact per capita or per unit of GDP. The role of standardization is to make the converted data comparable.

### The environmental and socioeconomic impacts of the global power sector transition

**Fig. 2:**
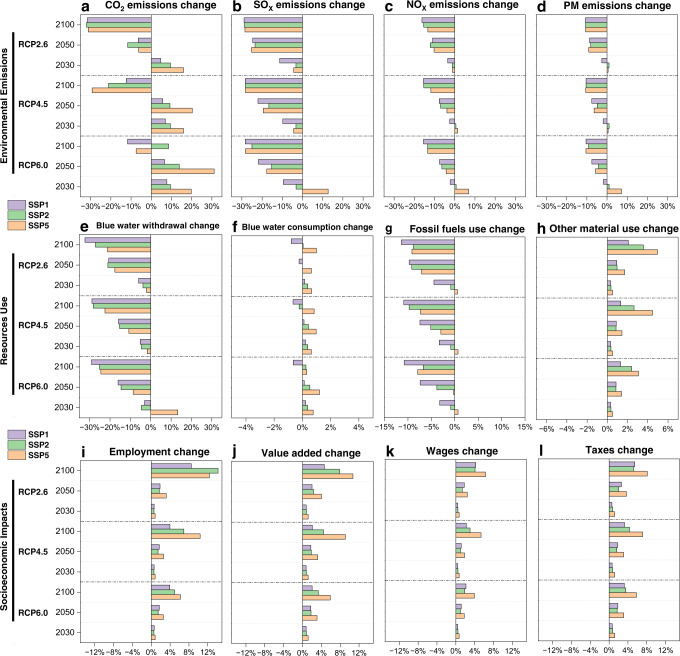
The percentage changes in global total environmental and socioeconomic impacts in 2030, 2050 and 2100 driven by the global power sector transition under nine different scenarios compared to those in 2015. **a** CO_2_ emission, **b** SO_X_ emissions, **c** NO_X_ emissions, **d** PM (PM_2.5_ and PM_10_), **e** blue water withdrawal (industry), **f** blue water consumption, **g** fossil fuels use, **h** other material use (biomass, metal, and nonmetal minerals), **i** employment, **j** value added, **k** wages, and **l** taxes. The nine different scenarios are SSP5 + RCP6.0, SSP5 + RCP4.5, SSP5 + RCP2.6 SSP2 + RCP6.0, SSP2 + RCP4.5, SSP2 + RCP2.6, SSP1 + RCP6.0, SSP1 + RCP4.5 and SSP1 + RCP2.6.

Figure 2 shows the percentage changes in global total environmental emissions, resource use, and socioeconomic impacts (the basic indicators used to evaluate SDG progress) driven by the global power transition under nine different scenarios: SSP5 + RCP6.0, SSP5 + RCP4.5, SSP5 + RCP2.6 SSP2 + RCP6.0, SSP2 + RCP4.5, SSP2 + RCP2.6, SSP1 + RCP6.0, SSP1 + RCP4.5 and SSP1 + RCP2.6.

We find that the global power transition has the strongest CO_2_ emission reduction effect under the RCP2.6 scenarios. For example, global CO_2_ emissions in 2015–2100 (34.85 Gt in 2015) would decrease by 31.17%, 32.09%, and 31.64% under SSP5 + RCP2.6, SSP2 + RCP2.6 and SSP1 + RCP2.6, respectively, which is much higher than those under the RCP6.0 and RCP4.5 scenarios (Fig. 2a). The discrepancy in emissions under different scenarios is mainly based on the assumptions of different energy mixes of electricity production (Supplementary Data 7). Given the stronger pollution controls in the future^47,48^, all the projections show decreasing trends of NO_X_ (Fig. 2b), SO_X_ (Fig. 2c), and PM (PM_2.5_ and PM_10_) emissions (Fig. 2d).

Our scenario analysis shows that blue water consumption will continue to grow by 2100 under the SSP5 and SSP2 scenarios, mainly due to the expansion of nuclear, biomass, or gas power, in contrast to the decrease in blue water consumption under the SSP1 scenarios (Fig. 2f). However, industrial blue water withdrawal would gradually decrease under all scenarios due to the extensive application of circulating cooling technology (Fig. 2e).

Along with higher demand for electricity in the future, all scenarios are accompanied by increasing use of materials, such as biomass, metal, and nonmetal minerals for power transitions (Fig. 2h), except for a decrease in fossil fuels (Fig. 2g). However, the power sector would consume much less fossil fuel and more biomass, metal, and nonmetal minerals under the RCP2.6 scenarios compared with the results under other scenarios.

In terms of the socioeconomic impacts of power production and the power transition, we could see a significant increase in employment, value-added, wages, and taxes (Fig. 2i–l) under all scenarios due to the high future demand for electricity. As the per unit of installed capacity of renewables can generate more jobs than that of coal power^49^, our results show that power generation and the power transition under the RCP2.6 scenarios (the most ambitious scenario with renewables generation) may bring more job opportunities compared with the results under the RCP6.0 and RCP4.5 scenarios.

### The impacts of the power transition on achieving global SDGs

Here, we translate the changes in environmental and socioeconomic indicators into global SDG progress using the United Nations SDG approach (see the Methods section). Our analysis shows that the global SDG index score, defined as the overall performance in achieving all individual SDG targets evaluated, would increase in the medium term and the long term under all scenarios. For example, the global SDG index score would increase from 54.70 in 2015 to 58.01 (6.05%) in 2050 and 61.33 (12.13%) in 2100 under SSP1 + RCP2.6 (Supplementary Table 1). Advances in technology and efficiency in electricity generation play a dominant role in the global SDG performance of the power sector. However, in the short term, the continued growth of fossil power may hinder global SDG progress. For example, the global SDG index score in 2030 would reach a lower level under the SSP5 scenarios, decreasing to 54.17 (−0.97%), 54.59 (−0.20%), and 54.60 (−0.19%) under SSP5 + RCP6.0, SSP5 + RCP4.5 and SSP5 + RCP2.6, respectively.

**Fig. 3:**
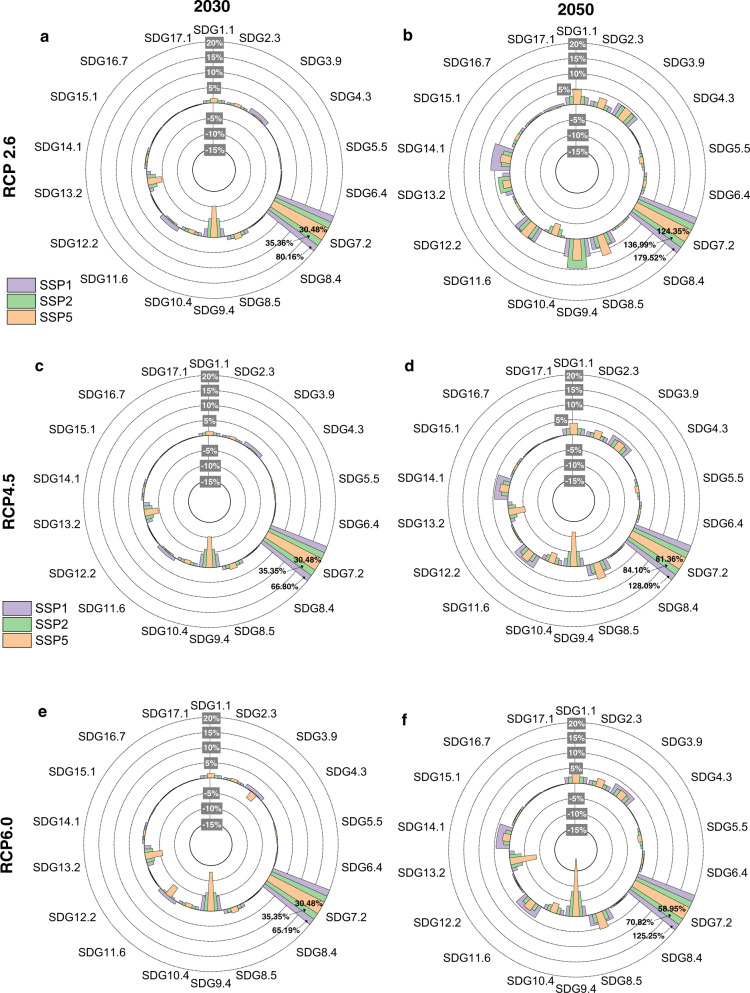
Global individual SDG target score changes in 2030 and 2050. RCP2.6 scenarios for **a** 2030 and **b** 2050, RCP4.5 scenarios for **c** 2030 and **d** 2050, and RCP6.0 scenarios for **e** 2030 and **f** 2050. Note: Global individual SDG target score changes in 2100 are shown in Supplementary Fig. 1. The individual SDG targets include SDG 1.1 (eradicate extreme poverty for all people everywhere), SDG 2.3 (enhance agricultural productive capacity), SDG 3.9 (reduce the number of deaths and illnesses from hazardous chemicals and air, water and soil pollution and contamination), SDG 4.3 (ensure equal access for all women and men to affordable and quality technical, vocational and tertiary education, including university), SDG 5.5 (ensure women’s full and effective participation and equal opportunities for leadership at all levels of decision-making in political, economic and public life), SDG 6.4 (ensure sustainable withdrawals and supply of freshwater), SDG 7.2 (increase substantially the share of renewable energy in the global energy mix), SDG 8.4 (improve resource efficiency in consumption and production), SDG 8.5 (achieve full and productive employment), SDG 9.4 (promote clean and sustainable industrialization), SDG 10.4 (adopt policies, especially fiscal, wage and social, protection policies, and progressively achieve greater equality), SDG 11.6 (reduce the adverse per capita environmental impact of cities), SDG 12.2 (achieve the sustainable management and efficient use of natural resources), SDG 13.2 (integrate climate change measures into national policies, strategies and planning), SDG 14.1 (prevent and significantly reduce marine pollution of all kinds, in particular from land-based activities, including marine debris and nutrient pollution), SDG 15.1 (ensure sustainable use of terrestrial ecosystems), SDG 16.7 (ensure responsive, inclusive, participatory and representative decision-making at all levels), and SDG 17.1 (strengthen domestic resource mobilization, including through international support to developing countries, to improve domestic capacity for tax and other revenue collection).

By 2030, most individual SDG target scores would not change significantly (Fig. 3). However, with the rapid expansion of renewable power, progress toward achieving SDG 7.2 (increase substantially the share of renewable energy in the global energy mix) increases significantly, with a range of 30.48% (SSP5 + RCP6.0) to 80.16% (SSP1 + RCP2.6). In contrast, global progress toward achieving SDG 9.4 (promote clean and sustainable industrialization) and SDG 13.2 (integrate climate change measures into national policies, strategies, and planning) would be stalled, mainly due to further growth in CO_2_ emissions from fossil electricity.

By 2050, progress toward achieving most individual SDG targets would accelerate under all scenarios (Fig. 3). SDG 7.2 is the top indicator of improvement, with a range of 58.95% (SSP5 + RCP6.0) to 179.52% (SSP1 + RCP2.6). The changes in progress toward achieving SDG 1.1 (eradicate extreme poverty for all people everywhere), SDG 2.3 (enhance agricultural productive capacity), SDG 3.9 (reduce the number of deaths and illnesses from hazardous chemicals and air, water and soil pollution and contamination), SDG 4.3 (ensure equal access for all women and men to affordable and quality technical, vocational and tertiary education, including university), SDG 6.4 (ensure sustainable withdrawals and supply of freshwater), SDG 8.4 (improve resource efficiency in consumption and production), SDG 8.5 (achieve full and productive employment), SDG 11.6 (reduce the adverse per capita environmental impact of cities), SDG 12.2 (achieve the sustainable management and efficient use of natural resources), SDG 14.1 (prevent and significantly reduce marine pollution of all kinds, in particular from land-based activities, including marine debris and nutrient pollution), and SDG 15.1 (ensure sustainable use of terrestrial ecosystems) are below or approximately 5%. However, the power transition will create more jobs for men, and the rate of increase in wages will be less than that in value-added, leading to a slight decline (less than 4%) in equality-related indicators, i.e., SDG 5.5 (ensure women’s full and effective participation and equal opportunities for leadership at all levels of decision making in political, economic and public life) and SDG 10.4 (adopt policies, especially fiscal, wage and social, protection policies, and progressively achieve greater equality). Looking forward to 2100, progress toward achieving most individual SDGs is more obvious (Supplementary Fig. 1).

### The impacts of the power transition on achieving regional SDGs

Among the various SSP + RCP combinations, we select three pathways, i.e., SSP1 + RCP2.6, SSP2 + SSP4.5, and SSP5 + RCP6.0, for projecting regional SDG index scores. The selected pathways do not cover all possible futures; rather, they represent different levels of power transition, including the business-as-usual (SSP2 + RCP4.5) pathway, renewable-based (SSP1 + RCP2.6) pathway, and fossil fuel-based (SSP5 + RCP6.0) pathway.

**Fig. 4:**
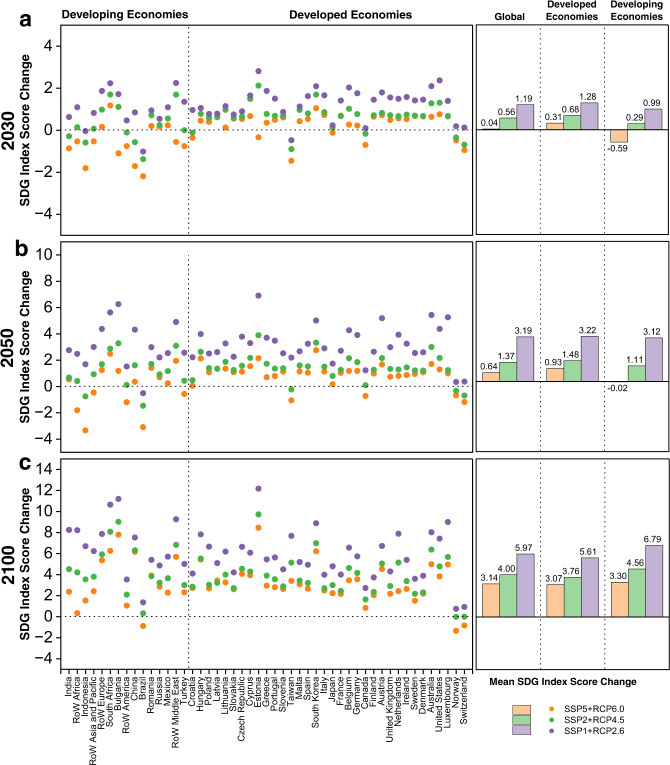
Changes in regional SDG index scores in 2030, 2050 and 2100 compared to those in 2015 under the SSP5 + RCP6.0, SSP2 + RCP4.5 and SSP1 + RCP2.6 scenarios. **a** changes in 2030, **b** changes in 2050, and **c** changes in 2100. Note: On the horizontal axis, the farther to the right an economy is, the higher its gross national income (GNI) per capita in the base year 2015. Using the World Bank’s classification based on income, we further classify 49 EXIOBASE economies into 34 developed economies (i.e., high-income economies) and 15 developing economies (Supplementary Table 2).

The changes in the SDG index score vary significantly across economies (Fig. 4). In general, the higher the GNI per capita is, the more inclined an economy is to improve its SDG index score in the short term and medium term, and vice versa. In either 2030 or 2050, the average level of SDG improvement in developing economies will be lower than that in developed economies under all scenarios. Under SSP5 + RCP6.0, developing economies will even experience a decline (0.59 in 2030, 0.02 in 2050) in the average SDG index score as they continue to use fossil fuels in large quantities. In the long term, the SDG improvement level of developing economies will exceed that of developed economies.

Some economies that currently rely on fossil fuels can also achieve SDG improvements in the short to medium term when they adopt rapid transition strategies. For example, Estonia, which is a country that completely relies on fossil fuels for power generation, would experience the largest increase in the SDG index score by 2050 under all scenarios, with a range of 2.15 (SSP5 + RCP6.0) to 6.91 (SSP1 + RCP2.6), due to significant expansion of renewable power to substitute for coal power under the European Climate Law^50^. This result verifies that strict climate legislation can effectively improve the sustainable development level of regions that are highly dependent on fossil power. In contrast, under SSP5 + RCP6.0, Indonesia, Brazil, and rest of the world (RoW) Africa will experiences declines of 3.34, 3.09, and 1.81, respectively, in their SDG index score because fossil fuel power would grow substantially in these emerging economies and climate change-related SDG (SDG 9.4 and SDG 13.2) progress would be hampered (Fig. 4a).

**Fig. 5:**
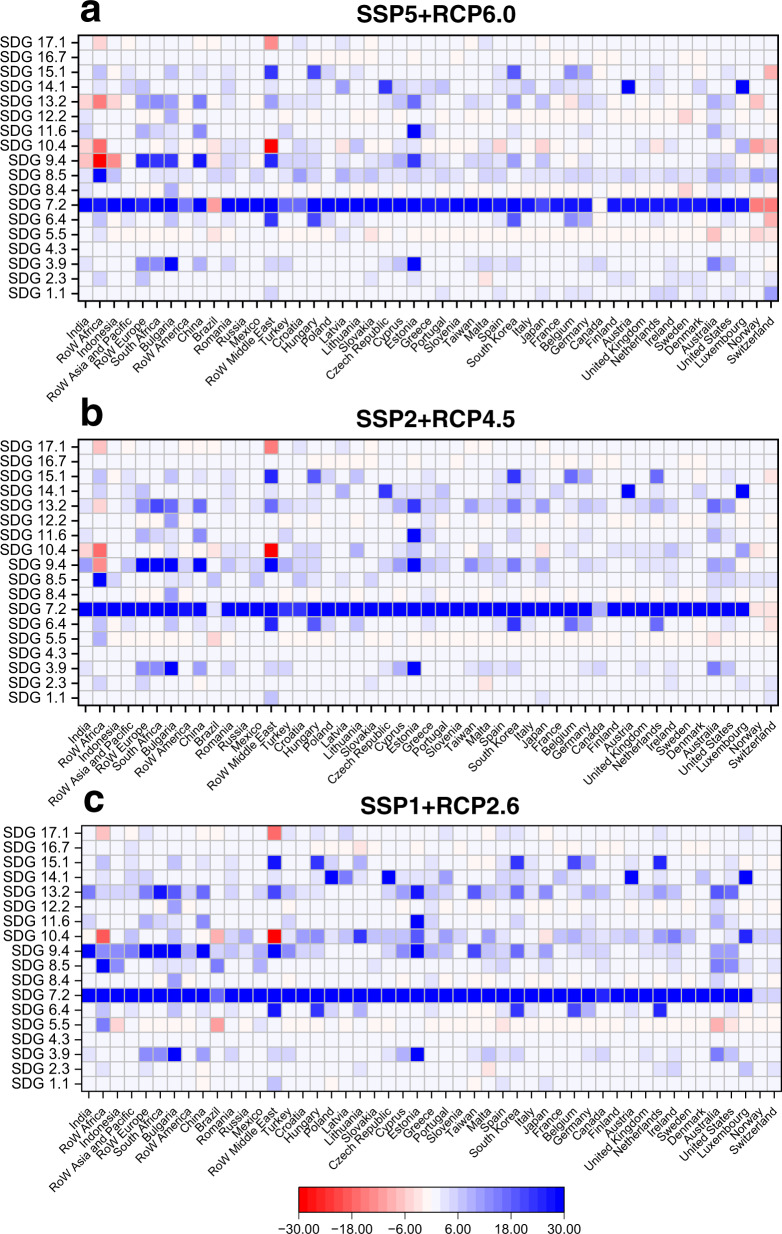
Changes in the regional individual SDG score between 2015 and 2100 under the SSP5 + RCP6.0, SSP2 + RCP4.5 and SSP1 + RCP2.6 scenarios. **a** SSP5 + RCP6.0, **b** SSP2 + RCP4.5, and **c** SSP1 + RCP2.6.

Regional power transitions could also lead to synergies and trade-offs between different individual SDGs (Fig. 5). Regarding synergies, scenario SSP1 + RCP2.6 shows that more than 90% of the economies considered would have an increase in more than 15 individual SDG scores by 2100 simultaneously. However, there are differences between some individual SDGs and the SDG index. For example, South Africa’s SDG index score would increase by 10.65 in 2100, but due to the increase in material use during the transition, its SDG 8.4 score would fall by 0.24. In addition, the power transition will improve all individual SDG scores in some economies, such as Romania and Mexico.

### The effects of power transition-related changes in international trade on achieving the SDGs

**Fig. 6:**
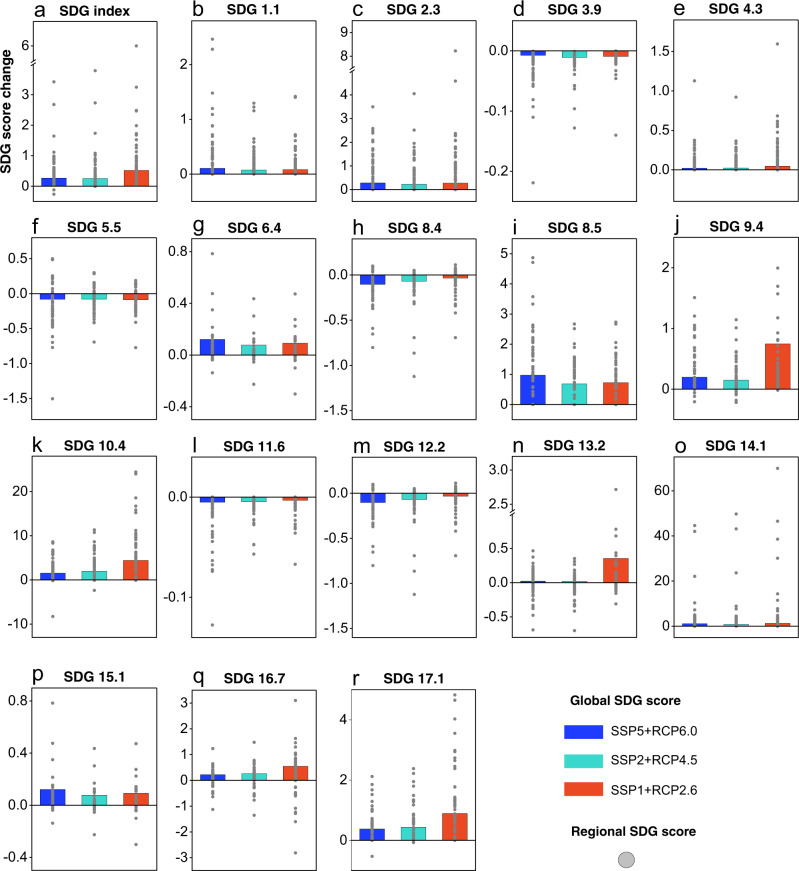
The impacts of power transition-related international trade on global and regional SDG performance between 2015 and 2100 under the SSP5 + RCP6.0, SSP2 + RCP4.5 and SSP1 + RCP2.6 scenarios. **a** SDG index, **b** SDG 1.1 (eradicate extreme poverty for all people everywhere), **c** SDG 2.3 (enhance agricultural productive capacity), **d** SDG 3.9 (reduce the number of deaths and illnesses from hazardous chemicals and air, water and soil pollution and contamination), **e** SDG 4.3 (ensure equal access for all women and men to affordable and quality technical, vocational and tertiary education, including university), **f** SDG 5.5 (ensure women’s full and effective participation and equal opportunities for leadership at all levels of decision-making in political, economic and public life), **g** SDG 6.4 (ensure sustainable withdrawals and supply of freshwater), **h** SDG 8.4 (improve resource efficiency in consumption and production), **i** SDG 8.5 (achieve full and productive employment), **j** SDG 9.4 (promote clean and sustainable industrialization), **k** SDG 10.4 (adopt policies, especially fiscal, wage and social, protection policies, and progressively achieve greater equality), **l** SDG 11.6 (reduce the adverse per capita environmental impact of cities), **m** SDG 12.2 (achieve the sustainable management and efficient use of natural resources), **n** SDG 13.2 (integrate climate change measures into national policies, strategies and planning), **o** SDG 14.1 (prevent and significantly reduce marine pollution of all kinds, in particular from land-based activities, including marine debris and nutrient pollution), **p** SDG 15.1 (ensure sustainable use of terrestrial ecosystems), **q** SDG 16.7 (ensure responsive, inclusive, participatory and representative decision-making at all levels), and **r** SDG 17.1 (strengthen domestic resource mobilization, including through international support to developing countries, to improve domestic capacity for tax and other revenue collection). Note: The impact of international trade on SDG 7.2 (increase substantially the share of renewable energy in the global energy mix) progress, cannot be quantified by MRIO, therefore, is not shown here.

Power transitions will not only change the scale and patterns of international trade but also exert effects on the environmental emissions, resource consumption, employment, value added, wages, and taxes embodied in exports and imports, thus influencing SDG performance in different regions. Power transition-induced changes in international trade would improve overall SDG performance (0.49–0.95%) globally between 2015 and 2100 (Fig. 6a). However, the overall impact would be rather limited, as the amount of traded commodities and services (measured by monetary value) related to the power sector accounts for only less than 2% of international trade. Socioeconomic-related SDG (SDG 10.4) performance would have the highest degree of improvement (2.28–6.55%), mainly due to the increase in wages embodied in renewable power-related trade (Fig. 6k). Employment-related SDG (SDG 8.5) performance would be improved, mainly because of the expansion of labor-intensive renewable power sectors (Fig. 6i). However, all scenarios would show a decline (0.04–0.13%) in the average scores of material use-related SDGs (SDGs 8.4 and 12.2) due to the increase in power production-related resource use met by international trade (Fig. 6h, m).

From a regional perspective, more than 70% of economies would improve their SDG performance under all scenarios (Fig. 6). However, the material use-related (SDGs 8.4 and 12.2) and environmental emissions-related SDGs (SDG 11.6) performance of developing economies with rich fossil energy and material resources, such as the those in the Middle East, would be impeded by international trade, as the expansion of power production results in an increase in the power sector-related resource consumption and environmental emissions embodied in international trade.

## Discussion

This study performs a quantitative analysis of the impacts of power sector transition on global and regional performance on achieving multiple SDGs. We find an improvement in global SDG index scores (the average score of 18 selected SDG targets) during the 2015–2100 period under all nine combination scenarios. The power transition brings increases of 4.42–7.40%, 7.55–8.93%, and 9.48–12.13% in global SDG index scores under the RCP6.0, RCP4.5 and RCP2.6 scenarios, respectively. However, the change in the regional individual SDG score is not always consistent with the change in the average SDG index score. For instance, for 15 out of 49 economies, the resource-related SDG (SDGs 8.4 and 12.2) scores will become worse if the current fossil-dominated power structure transitions to a renewables-dominated power structure (SSP1 + RCP2.6). Moreover, we conduct a sensitivity analysis to assess the sensitivity of the SDG scores to lower and upper bounds settings for normalization of indicator values. We select SDG scores change in 2100 as a proxy to present the results of our sensitivity analysis. Their detailed settings are shown in Supplementary Information, and the results are shown in Supplementary Fig. 2.

According to the Sustainable Development Report 2020, progress toward achieving the SDGs by 2030 lags far behind the schedule predesigned by the UN^29^. One of the main reasons is that there is a lack of understanding of the interactions between SDGs, which is essential to making trade-offs between the SDGs and advancing the achievement of the overall SDGs with minimal efforts^10,51^. Our research reveals the SDG synergies and trade-offs in global and regional power transitions, providing insights into advancing the power transformation and improving the current SDG “dilemma”.

Our results demonstrate that whether global SDG performance can be improved will be determined by developing economies’ power transition. The main reason is that fossil power contributes to meeting more than 70% of the electricity demand in developing economies. As a result of the gradual expansion of the population and economy, the electricity demand of developing economies will increase by 81.6–112.3% between 2015 and 2050, which is much higher than the increase in developed economies (23.2–28.4%). If power generation in developing economies is still dominated by fossil fuels, there will be a large amount of greenhouse gas and pollutant emissions, as well as a large amount of water resources, fossil fuel, and mineral depletion, thus posing great threats to global SDG progress.

Promoting the clean and low-carbon power transition in developing economies is crucial to global SDG progress. Meanwhile, due to the different levels of economic development and the different power structures, different developing economies need to take varying measures.

For Africa, the continent with the lowest income (gross national income) per capita, the greatest challenge it faces in making the power transition is the lack of sufficient financial support^52^. For example, the African low-carbon electricity transition cannot be achieved without investments in power growing by two and a half times through 2040 according to the International Energy Agency (IEA)^1^. Given the limited financial capacity and financial constraints of the utilities of governments, private sources of finance will be critical to bridge investment gaps. However, more than 1/3 of Sub-Saharan African countries, such as Nigeria and Sudan, do not allow for private sector participation in electricity generation or networks, which greatly jeopardizes the decarbonization of electricity in these areas^1^. For the smooth transition of these regions, private investment needs to be appropriately introduced to avoid financing gaps. In addition, blending international concessional capital with private capital has been proven to be particularly effective in Africa. Since 2015, blended finance has an average of approximately $9 billion annually around the world. Africa has accounted for the main transactions, attracting over 60% in 2020^53^.

For China and India, the two largest coal-fired power producers in the world, a rapid transition away from unabated coal use is essential. Recent regional trends reflect a shift in coal power prioritization from the US and the EU to many fast-developing countries in Asia, especially China, and India^54^. Thus, specific policy efforts that targeted coal-power production reduction are critical, for example, reductions in multilateral development banks’ financing of coal projects and national limits on coal consumption.

In addition, given that climate change is affecting the world as a whole and given that low-carbon technologies in most developing countries are still in their early stages, developed economies can consider supporting the power transition in developing economies through renewable power technology transfer, as most of the patents associated with renewable energies are privately owned^55^.

Transforming the power sector to low-carbon energy under RCP2.6 (or rapid low-carbon power transition) is verified to bring enormous co-benefits for global SDG performance overall. However, the situation differs from one region to another. The achievement of most individual SDGs in some economies, such as Germany and Spain, can be advanced by a rapid low-carbon power transition. This result indicates that the current and stated transition strategies of these countries are relatively sustainable. Notably, however, the power transition may lead to local SDG conflicts in some economies. For example, the Indian government’s clean energy transition strategies (solar capacity addition targets are accompanied by the retirement of thermal capacity) will create job opportunities primarily (60% of total) located in the western and southern parts of India (advancing the achievement of SDG 8.5: achieve full and productive employment), while leading to job losses being concentrated in the coal-mining states located in eastern India (hindering the achievement of SDG 8.5)^56^. Thus, a comprehensive review of the cross-regional impact of the power transition in large economies, such as the United States and India, is recommended to reduce regional imbalances from the transition. Meanwhile, specific development plans for subregional low-carbon power transitions are needed.

For most countries, a rapid low-carbon transition may cause conflicts between individual SDGs and progress toward achieving them (where progress toward achieving one goal hinders progress toward achieving another), which needs great attention. For example, the expansions of wind power and PV power in the United States will increase the demand for metals and nonmetals and undermine its achievement of SDG 8.4. In response to the material requirement or bottleneck for the future deployment of low-carbon power technologies, it is critical to increase the secondary supply of materials (recycling) instead of expanding mineral exploitation. Given the low rate of recycling of materials and the high recycling costs in the power sector^7^, more efforts with regard to the centralized recovery of retired electrical equipment and the development of technologies with lower costs and higher recovery rates need to be made.

Our results also indicate that the international trade associated with the low-carbon transition of the power sector has a limited effect on the average SDG performance at the global scale, but it may profoundly affect the SDG process of individual countries. This means that cross-national inequities in SDG progress may be exacerbated due to the expansion of renewable power or the reduction of fossil fuels in the electricity mix. For example, under the SSP2 + RCP2.6 scenario, by 2050, 42.13% of metal use increases (hindering the achievement of SDGs 8.4 and 12.2) in RoW America will be caused by the power transition in the country itself, and the remaining 57.87% will be driven by the ripple effects of the low-carbon transition in other countries (advancing the achievement of SDGs 9.4 and 13.2) through global supply chains. This result emphasizes the global systemic effects of the power transition, which calls for supply chain management when formulating power transition strategies to facilitate best practices in minimizing the impacts on achieving the SDGs.

Limitations and caveats apply to our study. First, our study is limited by the scope of our model and data. Although the EXIOBASE MRIO model and GCAM are diverse enough to cover the entire list of 17 SDGs and most of the key areas of sustainable development pertaining, they do not cover all systems (e.g., biodiversity and human health). Thus, our research does not capture the full complexity of interlinkages with the SDGs. Future research can further extend the model scope and data availability to explore the contribution of the power transition to SDG progress. Second, the nine selected scenarios in our study are used only as illustrative archetypes and are not intended to cover all future possibilities. Third, since we mainly focus on the impact of the power transition on SDG progress, the global trade patterns (from 2015) are assumed to remain the same for all scenarios and years to eliminate any possible confounding effects of changes in the global supply chain.

## Methods

### Scenarios of future power generation by region and technology

In this study, we derive future power transition pathways under a range of climate mitigation scenarios from the GCAM. The GCAM is a global model that simulates the behavior of and interactions between five systems: the energy system, water, agriculture and land use, the economy, and the climate. It has been widely used to produce scenarios for international and national assessments^26^. Market equilibrium is the core operating principle for the GCAM, which solves for a set of market prices so that supply and demand are balanced in all these markets across the model.

Nine scenarios are selected across two aspects to analyze future global power generation by region and technology based on reports by the Intergovernmental Panel Climate Change (IPCC)^57^. One aspect is the SSPs^36^, which were developed along the dimensions of challenges to mitigation and adaptation to climate change and can sufficiently cover the relevant socioeconomic dimensions. The other aspect is the RCPs^38^, which represents the ambition of climate policies. Each SSP + RCP combination (SSP5 + RCP6.0, SSP5 + RCP4.5, SSP5 + RCP2.6, SSP2 + RCP6.0, SSP2 + RCP4.5, SSP2 + RCP2.6, SSP1 + RCP6.0, SSP1 + RCP4.5, and SSP1 + RCP2.6.) represents an integrated scenario of future climate and societal change and can be used to investigate the global and regional power transition effort required to achieve that particular climate outcome. In detail, the GCAM directly provides the regional (32 regions globally), renewable, nuclear, fossil fuel and with/without carbon capture and storage (CCS)-specific power generation every 5 years from 2015 to 2100.

### Quantifying the environmental and socioeconomic impacts of the power transition

The MRIO model is used to quantify the environmental and socioeconomic impacts of the power transition. This model captures both the direct and indirect (supply chain) effects of ten power production subsectors (including coal power, gas power, nuclear power, hydroelectricity, wind power, petroleum and other oil derivatives power, biomass and waste power, solar PV, solar thermal, and geothermal power) on CO_2_ emissions, SO_X_ emissions, NO_X_ emissions, PM emissions (PM_2.5_ and PM_10)_, blue water withdrawal, blue water consumption, material use (fossil fuel, biomass, metal, and nonmetal), employment, value-added, tax and wages.

The basic framework of the MRIO model is as follows:

1 $${{{{{\bf{X}}}}}}={\left({{{{{\bf{I}}}}}}-{{{{{\bf{A}}}}}}\right)}^{-1}{{{{{\bf{F}}}}}}$$

where$\,{{{{{\bf{X}}}}}}={\left[{{{{{{\bf{X}}}}}}}_{i}^{r}\right]}_{n\times 1}$,$\,{{{{{{\bf{X}}}}}}}_{i}^{r}$ is the total output of the *i*th sector in region *r*. **I** is the identity matrix. ${{{{{\bf{A}}}}}}={\left[{{{{{{\bf{A}}}}}}}_{{ij}}^{{rs}}\right]}_{n\times n}$ is the technical coefficient matrix, ${{{{{{\bf{A}}}}}}}_{{ij}}^{{rs}}$ is given by ${{{{{{\bf{A}}}}}}}_{{ij}}^{{rs}}={{{{{{\bf{Z}}}}}}}_{{ij}}^{{rs}}/{{{{{{\bf{X}}}}}}}_{j}^{s}$, in which ${{{{{{\bf{Z}}}}}}}_{{ij}}^{{rs}}$ represents the monetary value flows from the *i*th sector in region *r* to the *j*th sector in region *s* and ${{{{{{\bf{X}}}}}}}_{j}^{s}$ is the total output of the *j*th sector in region *s*. ${\left({{{{{\bf{I}}}}}}-{{{{{\bf{A}}}}}}\right)}^{-1}$ is the Leontief inverted matrix (**L**). **F** is a column vector of the row sums of matrix ${{{{{\bf{Y}}}}}}={\left[{{{{{{\bf{Y}}}}}}}_{i}^{{rs}}\right]}_{n\times m}$, which is the final demand matrix, and ${{{{{{\bf{Y}}}}}}}_{i}^{{rs}}$ represents the final demand of region *s* for the goods and services of the *i*th sector from region *r*.

The direct impacts of power production subsectors can be calculated using the following equation:

2 $${{{{{{\bf{D}}}}}}}_{k}^{t}={{{{{{\bf{E}}}}}}}_{k}^{t}*{{{{{{\bf{G}}}}}}}_{k}^{t}$$

where ${{{{{{\bf{D}}}}}}}_{k}^{t}$ is the direct impacts of power production subsector *k* in year *t*,$\,{{{{{{\bf{E}}}}}}}_{k}^{t}$ is the direct impact intensity (the direct impact per unit total output) of subsector *k* in year *t*, and ${{{{{{\bf{G}}}}}}}_{k}^{t}$ is the total output of subsector *k* in year *t*. In addition, we assume that the direct impacts change proportionately to total output ${{{{{{\bf{G}}}}}}}_{k}$ and the direct impact intensity ${{{{{{\bf{E}}}}}}}_{k}$ between the modeled year *t* + 1 and the previous year *t* considered in the analysis given by the scenarios.

3 $${{{{{{\bf{D}}}}}}}_{k}^{t+1}=\left({{{{{{\bf{E}}}}}}}_{k}^{t+1}/{{{{{{\bf{E}}}}}}}_{k}^{t}\right)*\left({{{{{{\bf{G}}}}}}}_{k}^{t+1}/{{{{{{\bf{G}}}}}}}_{k}^{t}\right)\times {{{{{{\bf{D}}}}}}}_{k}^{t}$$

The indirect environmental and socioeconomic impacts of power production subsectors are evaluated based on the intermediate inputs from other sectors into the power sectors using the following equation:

4 $${{{{{{\bf{R}}}}}}}_{k}^{t}=\widehat{{{{{{{\bf{f}}}}}}}_{k}^{t}}{{{{{{\bf{L}}}}}}}^{t}\widehat{{{{{{{{\bf{X}}}}}}}^{{\prime} }}_{k}^{t}}$$

where ${{{{{{\bf{R}}}}}}}_{k}^{t}$ is the indirect impacts of power production subsector *k* in year *t*, ${{{{{{\bf{f}}}}}}}_{k}^{t}$ is a vector of the direct impact intensity for all economic sectors (set the direct impact intensity of power production subsector *k* to zero) in all regions in year *t*, and ${{{{{{\bf{L}}}}}}}^{t}$ is the Leontief inverted matrix in year *t*. Since we only need the output of power production subsectors, we create column vector ${{{{{{\bf{X}}}}}}}^{{\prime} }$, composed of zeros and ${{{{{{\bf{X}}}}}}}_{i}^{r}$ at the appropriate positions for power production subsector *k*. In addition, we assume that the indirect impacts change proportionately to total output ${{{{{{\bf{G}}}}}}}_{k}$ and the direct impact intensity$\,{{{{{{\bf{f}}}}}}}_{k}$ between the modeled year *t* + 1 and the previous year *t* considered in the analysis.

5 $${{{{{{\bf{R}}}}}}}_{k}^{t+1}=\left({{{{{{\bf{f}}}}}}}_{k}^{t+1}/{{{{{{\bf{f}}}}}}}_{k}^{t}\right)*\left({{{{{{\bf{G}}}}}}}_{k}^{t+1}/{{{{{{\bf{G}}}}}}}_{k}^{t}\right)*{{{{{{\bf{R}}}}}}}_{t}$$

In general, in our study, MRIO analysis involves three types of coefficients, including direct impact intensities, the Leontief inverted matrix, and the total output of power sectors. First, the three types of coefficients in base year 2015 are calculated based on the EXIOBASE 3 database. Second, we simulate the coefficient changes in future scenarios in combination with GCAM. We use the changes in the scale of electricity generation and the impact per unit of electricity generation in the GCAM to represent the changes in the total output of power sectors and direct impact intensities in the MRIO model, respectively. As predicting how global trade patterns will change falls beyond the scope of our study, following a previous study^58^, we do not make assumptions about variations in the Leontief inverted matrix. Finally, more information on the data inputs and coefficients has been added to the Supplementary Data (Supplementary Data 1-7).

### Translating the changes in environmental and socioeconomic indicators into SDG progress

#### Step 1: Indicator selection

The indicators selected for the SDGs in this study are from the Global Indicator Framework for Sustainable Development Goals^59^ developed by the United Nations’ Inter-Agency and Expert Group on SDG Indicators, two reports titled “Indicators and a Monitoring Framework for the Sustainable Development Goals”^29^ and “Sustainable Development Report 2020”^60^ published by the United Nations’ Sustainable Development Solutions Network, and a study entitled “Assessing progress towards sustainable development over space and time”^61^ published in Nature. We review all SDG indicators and select SDG indicators (Table 1) based on the following three criteria: (1) criterion 1 (relevance): the indicators are likely to be affected by the power transition; (2) criterion 2 (comparability): the indicators can be quantified across organizational levels and temporal scales; and (3) criterion 3 (data): the data for quantifying the indicators are available. In general, 18 targets and 27 indicators can be used to evaluate the SDG index, which covers all 17 SDGs. More information on indicator selection can be found in the Supplementary Information (Supplementary Table 3).

**Table 1:** Indicators selected for quantifying the impacts of the power transition on achieving SDGs

SDGs	Targets	Indicators illustration
Goal 1. No poverty	1.1 Eradicate extreme poverty for all people everywhere	1.1.1 GDP per capita
Goal 2. Zero hunger	2.3 Enhance agricultural productive capacity	2.3.1 Agricultural value added per capita
Goal 3. Good health and well-being	3.9 Reduce the number of deaths and illnesses from hazardous chemicals and air, water, and soil pollution and contamination	3.9.1 SO_X_ emissions of per capita
		3.9.2 NO_X_ emissions of per capita
		3.9.3 PM (PM_2.5_ and PM_10_) emissions of per capita
Goal 4. Quality education	4.3 Ensure equal access for all women and men to affordable and quality technical, vocational, and tertiary education, including university	4.3.1 Education services size of per capita
Goal 5. Gender equality	5.5 Ensure women’s full and effective participation and equal opportunities for leadership at all levels of decision-making in political, economic, and public life	5.5.1 Ratio of male to female employment rate
Goal 6. Clean water and sanitation	6.4 Ensure sustainable withdrawals and supply of freshwater	6.4.1 Water-use efficiency: blue water consumption per GDP
		6.4.2 Level of water stress: blue water withdrawal (industry) as a proportion of available freshwater resources
Goal 7. Affordable and clean energy	7.2 Increase substantially the share of renewable energy in the global energy mix	7.2.1 Renewable energy share in the power generation
Goal 8. Decent work and economic growth	8.4 Improve resource efficiency in consumption and production	8.4.1 Domestic material use per capita: metal use, non-metallic minerals use, fossil fuels use and biomass use per capita
		8.4.2 Domestic material use per GDP: metal use, non-metallic minerals use, fossil fuels use and biomass use per GDP
	8.5 Achieve full and productive employment	8.5.1 Unemployment rate
Goal 9. Industry, innovation, and infrastructure	9.4 Promote clean and sustainable industrialization	9.4.1 CO_2_ emissions per unit of value added
Goal 10. Reduced inequalities	10.4 Adopt policies, especially fiscal, wage, and social, protection policies, and progressively achieve greater equality	10.4.1 Labor share of GDP
Goal 11. Sustainable cities and communities	11.6 Reduce the adverse per capita environmental impact of cities	11.6.1 Annual mean levels of fine particulate matter (e.g. PM_2.5_ and PM_10_) in cities (population weighted)
Goal 12. Responsible consumption and production	12.2 Achieve the sustainable management and efficient use of natural resources	12.2.1 Domestic material use per capita: metal use, non-metallic minerals use, fossil fuels use, and biomass use per capita
		12.2.2 Domestic material use per GDP: metal use, non-metallic minerals use, fossil fuels use, and biomass use per GDP
Goal 13. Climate change	13.2 Integrate climate change measures into national policies, strategies, and planning	13.2.1 CO_2_ emissions intensity of forest areas
		13.2.2 CO_2_ emissions intensity per capita
		13.2.3 CO_2_ emissions intensity per GDP
Goal 14. Life below water	14.1 Prevent and significantly reduce marine pollution of all kinds, in particular from land-based activities, including marine debris and nutrient pollution	14.1.1 NO_X_ emissions intensity of sea transport
		14.1.2 SO_X_ emissions intensity of sea transport
Goal 15. Life on land	15.1 Ensure sustainable use of terrestrial ecosystems	15.1.1 Water-use efficiency: blue water consumption per GDP
		15.1.2 Level of water stress: blue water withdrawal (industry) as a proportion of available freshwater resources
Goal 16. Peace, justice, and strong institutions	16.7 Ensure responsive, inclusive, participatory, and representative decision-making at all levels	16.7.1 Proportions of female in public institutions
Goal 17. Partnerships for the goals	17.1 Strengthen domestic resource mobilization, including through international support to developing countries, to improve domestic capacity for tax and other revenue collection	17.1.1 The percentage share of tax revenues in GDP

#### Step 2: Bound selection

Using 2015 as the baseline year, we calculate the score of the selected SDG indicators for all 49 countries/regions in EXIOBASE 3. The procedure consists of the following steps: To ensure data comparability across different SDG indicators, each indicator data point is rescaled from 0 to 100, with 0 indicating the worst performance and 100 denoting the optimum performance. Given that rescaling is very sensitive to extreme (outlier) values on both tails of the data distribution, we follow the methods proposed by the Sustainable Development Report 2020 to determine the upper bound and lower bound of each SDG indicator. We define the data at the bottom 2.5^th^ percentile of all economies’ SDG indicator performances for a given SDG indicator as the minimum value (0) and the data at the upper 2.5th percentile as the maximum value (100) for normalization to remove the effect of extreme values. This bound selection method is consistent with the approach recommended by the Organization for Economic Cooperation and Development (OECD) for comparing indicator performances and has been used by SDG research articles and the Sustainable Development Report^60^. In addition, we use relevant absolute quantitative thresholds for some SDG indicators, such as “zero emissions” and “absolute gender equality”.

#### Step 3: Normalization of indicator values

After determining the upper and lower bounds, we rescale the selected SDG indicator values across economies to a scale of 0 to 100 using Eq. (6):

6 $${Z}^{{\prime} }=\frac{Z-\min \left(Z\right)}{\max \left(Z\right)-{{\min }}(Z)}$$

where *Z* represents the raw data value for a given SDG indicator. Min and max are the bounds for the worst and best performance, respectively. ${Z}^{{\prime} }$ denotes the normalized value for a given SDG indicator.

#### Step 4: Calculation of SDG index scores

We calculate global and regional SDG index scores with the arithmetic mean of the individual SDG target scores. Following the approach used in the Sustainable Development Report 2020^60^ and previous research^24^, all 18 SDG targets are weighted equally in producing the aggregate measure since there is no a priori reason to give one measure greater weight than another. In addition, we conduct a sensitivity analysis to assess the sensitivity of the SDG index scores to lower and upper bound settings for normalization of the indicator values. The objective of our sensitivity analysis is to confirm that our conclusions are robust to the bound settings.

## Supplementary information

Supplementary Information

Description of Additional Supplementary Files

Supplementary Data 1

Supplementary Data 2

Supplementary Data 3

Supplementary Data 4

Supplementary Data 5

Supplementary Data 6

Supplementary Data 7

## Data Availability

The data generated in this study are provided in Supplementary Information, Supplementary Data and Source Data file. The data used for MRIO analysis are from EXIOBASE database (https://www.exiobase.eu/). The input data for scenario analysis are provided in Supplementary Data and also available on Zenodo: 10.5281/zenodo.7904808^62^. Source data are provided with this paper.

## Source data

Source Data

## Code availability

Codes for this work can be acquired at 10.5281/zenodo.7904808^62^.
