# Supplementary material for: The global power sector’s low-carbon transition may enhance sustainable development goal achievement
Source: Nat Commun. 2023 May 30;14:3144. doi: 10.1038/s41467-023-38987-4 (PMC10229651; doi:10.1038/s41467-023-38987-4)
Supplement: Supplementary file 1 — Supplementary Information [file 41467_2023_38987_MOESM1_ESM.pdf]

Supplementary Information for

The global power sector's low-carbon transition may enhance sustainable development goal achievement

Kun Peng[1], Kuishuang Feng[2], Bin Chen[3], Yuli Shan[4], Ning Zhang[1], Peng Wang[5], Kai Fang[6], Yanchao Bai[7], Xiaowei Zou[1], Wendong Wei[8], Xinyi Geng[9], Yiyi Zhang[10], Jiashuo Li[1,*]

[1] Institute of Blue and Green Development, Shandong University, Weihai 264209, China

[2] Department of Geographical Sciences, University of Maryland, College Park, MD 20742, United States

[3] Fudan Tyndall Center, Department of Environmental Science and Engineering, Fudan University, Shanghai 200438, China

[4] School of Geography, Earth and Environmental Sciences, University of Birmingham, Birmingham B15 2TT, UK

[5] Key Lab of Urban Environment and Health, Institute of Urban Environment, Chinese Academy of Sciences, Xiamen 361021, China

[6] School of Public Affairs, Zhejiang University, Hangzhou 310058, China

[7] College of Environmental Science and Engineering, Yangzhou University, Yangzhou 225127, China

[8] School of International and Public Affairs, Shanghai Jiao Tong University, Shanghai 200030, China

[9] Economics and Management School, Wuhan University, Wuhan 430070, China

[10] Guangxi Key Laboratory of Intelligent Control and Maintenance of Power Equipment, Guangxi University, Nanning 530004, China

* Correspondence: lijiashuo@sdu.edu.cn (J.L.).

Supplementary Notes. S1-S2

Supplementary Tables. 1-3

Supplementary Figures. 1-3

Supplementary Notes

Note S1. Robustness and sensitivity to lower and upper bounds settings

We conduct a sensitivity analysis to assess the sensitivity of the SDG scores to lower and upper bounds settings for normalization of indicator values. The objective of our sensitivity analysis is to confirm that our conclusions are robust to bounds settings.

We select global SDG scores change in 2100 as a proxy to present the results of our sensitivity analysis. The modified condition is that we increase or decrease the value of bounds by 10%. The sensitivity analysis results are shown in Supplementary Figure. 2.

We find that the sensitivity of the SDG index score (the overall performance in achieving all individual SDG evaluated) to value changes of bounds is with in 0.02, suggesting a 10% increase or dearease in the value of bounds can only change 0.2% of the SDG index score. In view of this, we believe that the SDG index is not sensitive to the change of the value of bounds.

Note S2 Comparison with the results of previous studies

We have reviewed recent studies for results comparison (select SSP2+RCP2.6 as a proxy) on global power generation pathways as modelled by five structurally different Integrated Assessment Modelling systems (GCAM, IMAGE, MESSAGE-GLOBIOM, POLES and REMIND)[1-4]. These large divergences between those projections in view of viable technology options for low-carbon electricity supply. For instance, some pathways rely heavily on nuclear or carbon capture and storage (CCS), while others focus mostly on renewable energy sources. In general, we find that our projections of 2050 electricity generation and electricity mix are within reasonable ranges (Supplementary Figure. 3).

Supplementary Tables

Supplementary Table 1. The percentage changes in global SDG index score in 2030, 2050, and 2100 under nine scenarios to that in 2015.

Scenarios	2030	2050	2100
SSP5+RCP6.0	-0.97%	0.32%	6.06%
SSP2+RCP6.0	0.26%	1.42%	4.42%
SSP1+RCP6.0	1.25%	3.60%	7.40%
SSP5+RCP4.5	-0.20%	1.62%	8.73%
SSP2+RCP4.5	0.26%	2.08%	8.93%
SSP1+RCP4.5	1.34%	3.74%	7.55%
SSP5+RCP2.6	-0.19%	4.66%	9.48%
SSP2+RCP2.6	0.26%	4.98%	11.89%
SSP1+RCP2.6	1.85%	6.05%	12.13%

Supplementary Table 2. GNI per capita for each economy and the economy classifications.

NO.	Country/region	GNI* per capita	Income group	Country group
1	Austria	43761	High Income	Developed economies
2	Belgium	41589	High Income	Developed economies
3	Bulgaria	6854	Upper middle income	Developing economies
4	Cyprus	16979	High Income	Developed economies
5	Czech Republic	16607	High Income	Developed economies
6	Germany	42040	High Income	Developed economies
7	Denmark	54890	High Income	Developed economies
8	Estonia	17020	High Income	Developed economies
9	Spain	25737	High Income	Developed economies
10	Finland	43223	High Income	Developed economies
11	France	37419	High Income	Developed economies
12	Greece	18069	High Income	Developed economies
13	Croatia	11985	High Income	Developed economies
14	Hungary	12112	High Income	Developed economies
15	Ireland	47666	High Income	Developed economies
16	Italy	30033	High Income	Developed economies
17	Lithuania	13749	High Income	Developed economies
18	Luxembourg	67868	High Income	Developed economies
19	Latvia	13722	High Income	Developed economies
20	Malta	23544	High Income	Developed economies
21	Netherlands	45230	High Income	Developed economies
22	Poland	12120	High Income	Developed economies
23	Portugal	18734	High Income	Developed economies
24	Romania	8875	Upper middle income	Developing economies
25	Sweden	51957	High Income	Developed economies
26	Slovenia	20246	High Income	Developed economies
27	Slovakia	15942	High Income	Developed economies
28	United Kingdom	44313	High Income	Developed economies
29	United States	58180	High Income	Developed economies
30	Japan	36336	High Income	Developed economies
31	China	8028	Upper middle income	Developing economies
32	Canada	42928	High Income	Developed economies
33	South Korea	28822	High Income	Developed economies
34	Brazil	8628	Upper middle income	Developing economies
35	India	1587	Lower middle income	Developing economies
36	Mexico	9368	Upper middle income	Developing economies
37	Russia	9200	Upper middle income	Developing economies
38	Australia	55555	High Income	Developed economies
39	Switzerland	85820	High Income	Developed economies
40	Turkey	10883	Upper middle income	Developing economies
41	Chinese Taiwan	23108	High Income	Developed economies
42	Norway	77446	High Income	Developed economies
43	Indonesia	3221	Lower middle income	Developing economies
44	South Africa	6116	Upper middle income	Developing economies
45	RoW Asia and Pacific	3357	Lower middle income	Developing economies
46	RoW America	6994	Upper middle income	Developing economies
47	RoW Europe	3546	Lower middle income	Developing economies
48	RoW Africa	1764	Lower middle income	Developing economies
49	RoW Middle East	10348	Upper middle income	Developing economies

Note: Using the World Bank's classification based on income[5], we further classify 49 EXIOBASE economies into 34 developed economies (i.e., high income economies) and 15 developing economies (i.e., middle and low income economies). * GNI (Gross National Income, US$) per capita in the base year 2015.

Supplementary Table 3. Whittling the 169 SDG targets by each criterion * for quantifying the impacts of power transition on SDGs.

NO.	Targets	Description
	**Goal 1. No poverty**	
1	1.1 Eradicate extreme poverty for all people everywhere	Meeting the three criteria<br>Selected Indicator: GDP per capita
2	1.2	Failing Criterion 3 (Data)
3-7	1.3-1.5, 1.a and 1.b	Failing Criterion 1 (Relevance)
	**Goal 2. Zero hunger**	
8, 9	2.1 and 2.2	Failing Criterion 1 (Relevance)
10	2.3 Double the agricultural productivity and incomes of small-scale food producers, in particular women, indigenous peoples, family farmers, pastoralists and fishers, including through secure and equal access to land, other productive resources and inputs, knowledge, financial services, markets and opportunities for value addition and non-farm employment	Meeting the three criteria<br>Selected Indicator:<br>Agricultural value added per capita
11-15	2.4 and 2.5, 2.a-2.c	Failing Criterion 1 (Relevance)
	**Goal 3. Good health and well-being**	
16-23	3.1-3.8	Failing Criterion 1 (Relevance)
24	3.9 Substantially reduce the number of deaths and illnesses from hazardous chemicals and air, water and soil pollution and contamination	Meeting the three criteria<br>Selected Indicators:<br>SO_X emissions of per capita;<br>NO_X emissions of per capita;<br>PM emissions of per capita
25-28	3.a-3.d	Failing Criterion 1 (Relevance)
	**Goal 4. Quality education**	
29, 30	4.1 and 4.2	Failing Criterion 1 (Relevance)

31	4.3 Ensure equal access for all women and men to affordable and quality technical, vocational and tertiary education, including university	Meeting the three criteria<br>Selected Indicator:<br>Education services size of per capita
32-38	4.4-4.7, 4.a-4.c	Failing Criterion 1 (Relevance)
	**Goal 5. Gender equality**	
39-42	5.1-5.4	Failing Criterion 1 (Relevance)
43	5.5 Ensure women's full and effective participation and equal opportunities for leadership at all levels of decision-making in political, economic and public life	Meeting the three criteria<br>Selected Indicator:<br>Ratio of male to female employment rate
44-47	5.6, 5.a-5.c	Failing Criterion 1 (Relevance)
	**Goal 6. Clean water and sanitation**	
48-50	6.1-6.3	Failing Criterion 1 (Relevance)
51	6.4 Substantially increase water-use efficiency across all sectors and ensure sustainable withdrawals and supply of freshwater to address water scarcity and substantially reduce the number of people suffering from water scarcity	Meeting the three criteria<br>Selected Indicators:<br>blue water consumption per GDP;<br>blue water withdrawal (industry) as a proportion of available freshwater resources
52-55	6.5 and 6.6, 6.a and 6.b	Failing Criterion 1 (Relevance)
	**Goal 7. Affordable and clean energy**	
56	7.1	Failing Criterion 3 (Data)
57	7.2 Increase substantially the share of renewable energy in the global energy mix	Meeting the three criteria<br>Selected Indicator:<br>Renewable energy share in the power generation
58, 59	7.3 and 7.a	Failing Criterion 1 (Relevance)

60	7.b	Failing Criterion 2 (Comparability)
	**Goal 8. Decent work and economic growth**	
61, 62	8.1 and 8.2	Failing Criterion 1 (Relevance)
63	8.3	Failing Criterion 3 (Data)
64	8.4 Improve progressively, through 2030, global resource efficiency in consumption and production and endeavour to decouple economic growth from environmental degradation, in accordance with the 10-Year Framework of Programmes on Sustainable Consumption and Production, with developed countries taking the lead	Meeting the three criteria<br>Selected Indicators:<br>Domestic material use per capita;<br>Domestic material use per GDP
65	8.5 Achieve full and productive employment and decent work for all women and men, including for young people and persons with disabilities, and equal pay for work of equal value	Meeting the three criteria<br>Selected Indicator:<br>Unemployment rate
66-72	8.6-8.10, 8.a and 8.b	Failing Criterion 1 (Relevance)
	**Goal 9. Industry, innovation, and infrastructure**	
73	9.1	Failing Criterion 1 (Relevance)
74, 75	9.2 and 9.3	Failing Criterion 3 (Data)
76	9.4 Upgrade infrastructure and retrofit industries to make them sustainable, with increased resource-use efficiency and greater adoption of clean and environmentally sound technologies and industrial processes, with all countries taking action in accordance with their respective capabilities	Meeting the three criteria<br>Selected Indicator:<br>CO_2 emissions per unit of value added
77-80	9.5, 9.a-9.c	Failing Criterion 1 (Relevance)
	**Goal 10. Reduced inequalities**	
81-83	10.1-10.3	Failing Criterion 1 (Relevance)
84	10.4 Adopt policies, especially fiscal, wage and social protection policies, and progressively achieve greater equality	Meeting the three criteria<br>Selected Indicator:<br>Labour share of GDP
85-90	10.5-10.7, 10.a-10.c	Failing Criterion 1 (Relevance)

	**Goal 11. Sustainable cities and communities**	
91-95	11.1-11.5	Failing Criterion 1 (Relevance)
96	11.6 Reduce the adverse per capita environmental impact of cities, including by paying special attention to air quality and municipal and other waste management	Meeting the three criteria<br>Selected Indicator:<br>Annual mean levels of fine particulate matter
97-100	11.7, 11.a-11.c	Failing Criterion 1 (Relevance)
	**Goal 12. Responsible consumption and production**	
101	12.1	Failing Criterion 1 (Relevance)
102	12.2 Achieve the sustainable management and efficient use of natural resources	Meeting the three criteria<br>Selected Indicators:<br>Domestic material use per capita;<br>Domestic material use per GDP
103-111	12.3-12.8, 11.a-11.c	Failing Criterion 1 (Relevance)
	**Goal 13. Climate change**	
112	13.1	Failing Criterion 1 (Relevance)
113	13.2 Integrate climate change measures into national policies, strategies and planning	Meeting the three criteria<br>Selected Indicators:<br>CO_2 emissions intensity of forest areas;<br>CO_2 emissions intensity per capita;<br>CO_2 emissions intensity per GDP
114-116	13.3, 13.a and 13.b	Failing Criterion 1 (Relevance)
	**Goal 14. Life below water**	

117	14.1 Prevent and significantly reduce marine pollution of all kinds, in particular from land-based activities, including marine debris and nutrient pollution	Meeting the three criteria<br>Selected Indicators:<br>NO_X emissions intensity of sea transport<br>SO_X emissions intensity of sea transport
118-126	14.2-14.7, 14.a-14.c	Failing Criterion 1 (Relevance)
	**Goal 15. Life on land**	
127	15.1 Ensure the conservation, restoration and sustainable use of terrestrial and inland freshwater ecosystems and their services, in particular forests, wetlands, mountains and drylands, in line with obligations under international agreements	Meeting the three criteria<br>Selected Indicator:<br>blue water withdrawal (industry) as a proportion of available freshwater resources
128	15.2	Failing Criterion 1 (Relevance)
129, 130	15.3 and 15.4	Failing Criterion 3 (Data)
131-138	15.5-15.9, 15.a-15.c	Failing Criterion 1 (Relevance)
	**Goal 16. Peace, justice, and strong institutions**	
139-144	16.1-16.6	Failing Criterion 1 (Relevance)
145	16.7 Ensure responsive, inclusive, participatory and representative decision-making at all levels	Meeting the three criteria<br>Selected Indicator:<br>Proportions of female in public institutions
146-150	16.8-16.10, 16.a and 16.b	Failing Criterion 1 (Relevance)
	**Goal 17. Partnerships for the goals**	
151	17.1 Strengthen domestic resource mobilization, including through international support to developing countries, to improve domestic capacity for tax and other revenue collection	Meeting the three criteria<br>Selected Indicator:<br>The percentage share of tax revenues in GDP
152-169	17.2-17.19	Failing Criterion 1 (Relevance)

Note: The indicators in this study are selected from the Global Indicator Framework for Sustainable Development Goals[6] developed by the United Nations' Inter-Agency and Expert Group on SDG Indicators, two reports titled "Indicators and a Monitoring Framework for the Sustainable Development Goals"[7] and "Sustainable Development Report 2020"[8] published by the United Nations' Sustainable Development Solutions Network, and a study entitled "Assessing progress towards sustainable development over space and time"[9] published in Nature. Three criteria [*] are used to determine alternative indicators for measuring SDG index: (1) Criterion 1 (Relevance): the indicators are likely to be affected by power transition, (2) Criterion 2 (Comparability): the indicators can be quantified across organizational levels and temporal scales, and (3) Criterion 3 (Data): the data for quantifying the indicators are available. In general, 18 targets and 27 indictors can be used to evaluate SDG index, which cover all the 17 SDGs.

Supplementary Figures

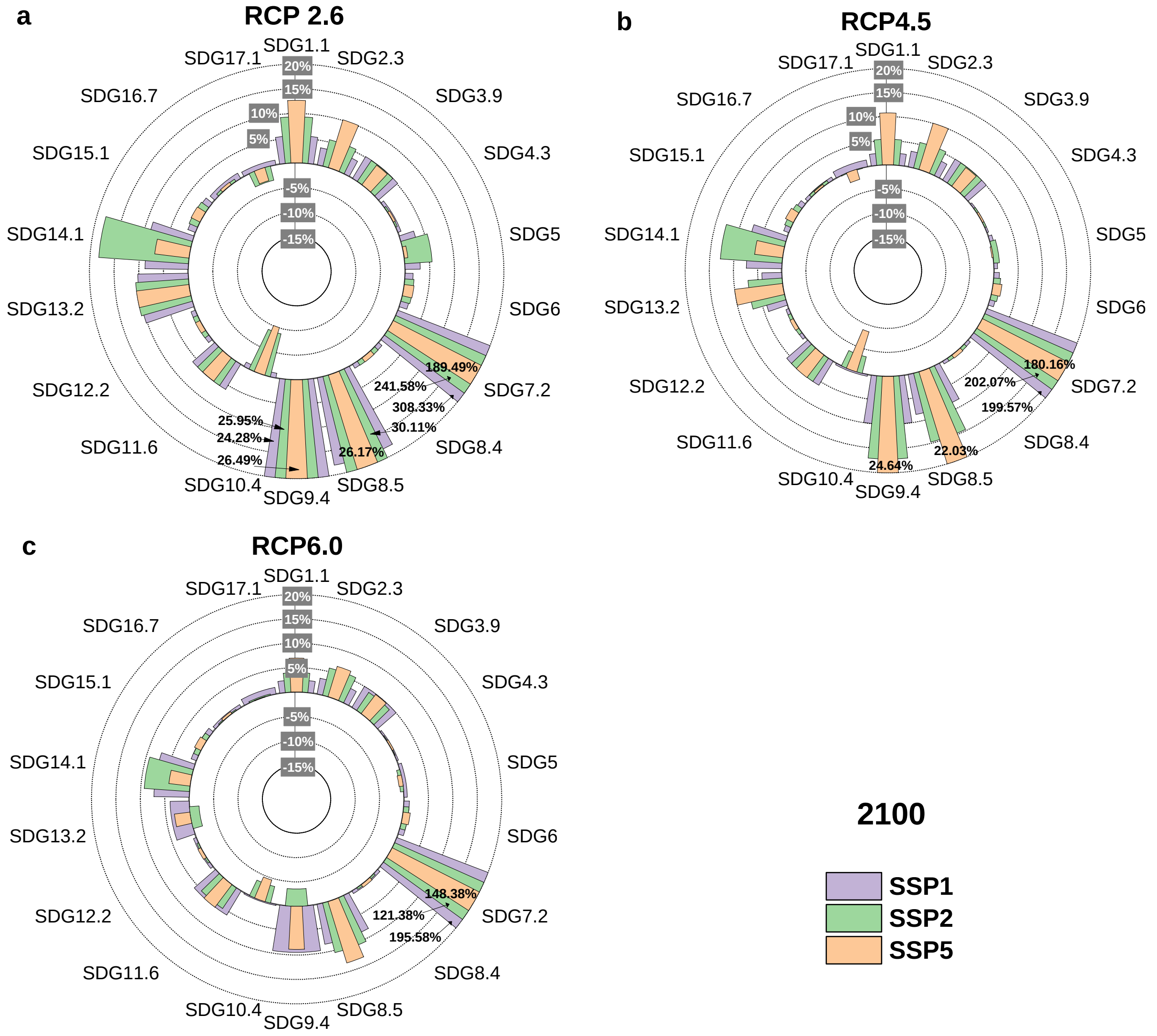

Supplementary Figure. 1 Global individual SDG target score changes in 2100. a RCP2.6 scenarios, **b** RCP4.5 scenarios, and **c** RCP6.0 scenarios. The individual SDG targets include SDG 1.1 (eradicate extreme poverty for all people everywhere), SDG 2.3 (enhance agricultural productive capacity), SDG 3.9 (reduce the number of deaths and illnesses from hazardous chemicals and air, water and soil pollution and contamination), SDG 4.3 (ensure equal access for all women and men to affordable and quality technical, vocational and tertiary education, including university), SDG 5.5 (ensure women's full and effective participation and equal opportunities for leadership at all levels of decision-making in political, economic and public life), SDG 6.4 (ensure sustainable withdrawals and supply of freshwater), SDG 7.2 (increase substantially the

share of renewable energy in the global energy mix), SDG 8.4 (improve resource efficiency in consumption and production), SDG 8.5 (achieve full and productive employment), SDG 9.4 (promote clean and sustainable industrialization), SDG 10.4 (adopt policies, especially fiscal, wage and social, protection policies, and progressively achieve greater equality), SDG 11.6 (reduce the adverse per capita environmental impact of cities), SDG 12.2 (achieve the sustainable management and efficient use of natural resources), SDG 13.2 (integrate climate change measures into national policies, strategies and planning), SDG 14.1 (prevent and significantly reduce marine pollution of all kinds, in particular from land-based activities, including marine debris and nutrient pollution), SDG 15.1 (ensure sustainable use of terrestrial ecosystems), SDG 16.7 (ensure responsive, inclusive, participatory and representative decision-making at all levels), and SDG 17.1 (strengthen domestic resource mobilization, including through international support to developing countries, to improve domestic capacity for tax and other revenue collection).

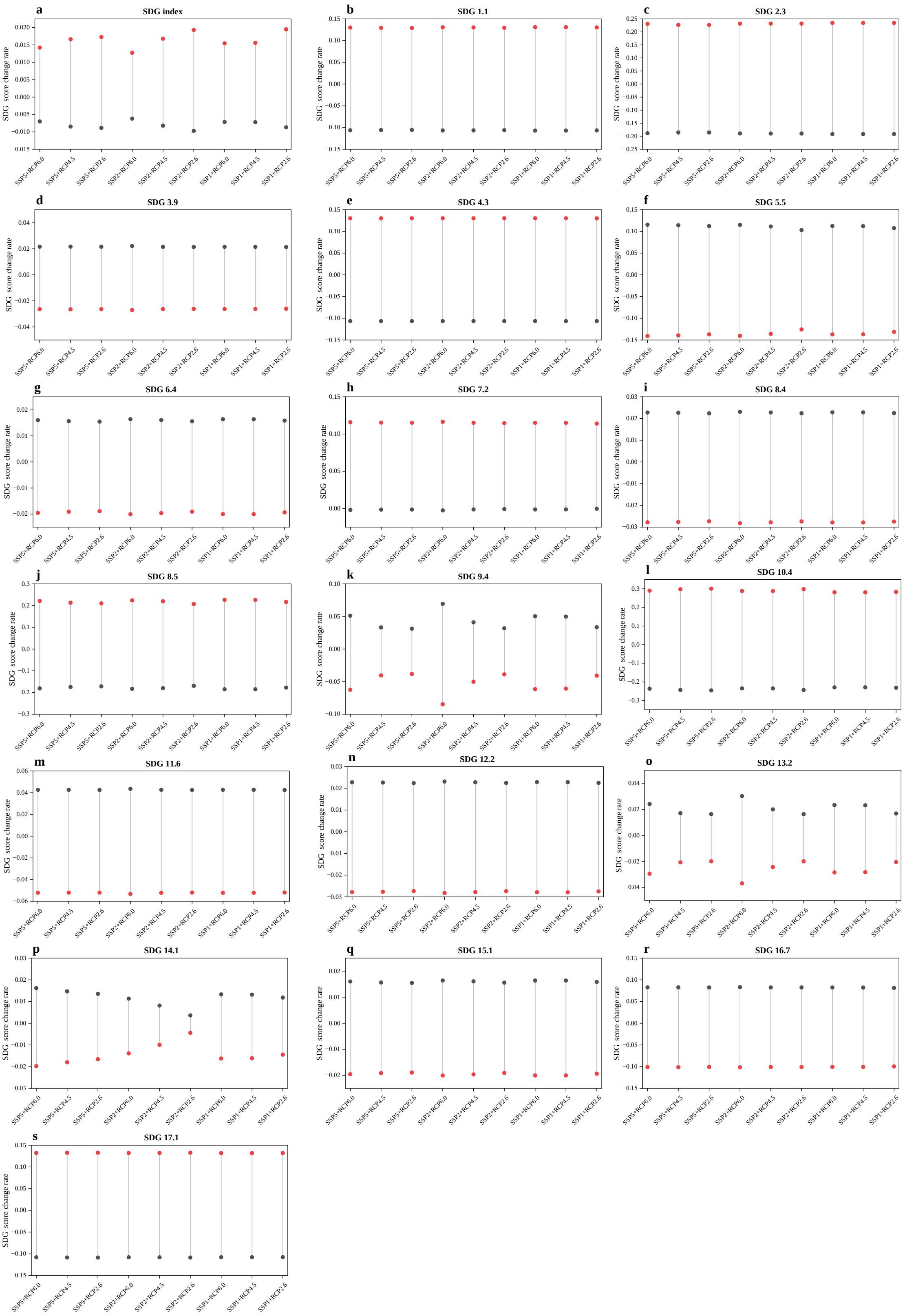

Supplementary Figure. 2 The impact of bounds settings for normalization of indicator values on global SDG scores.

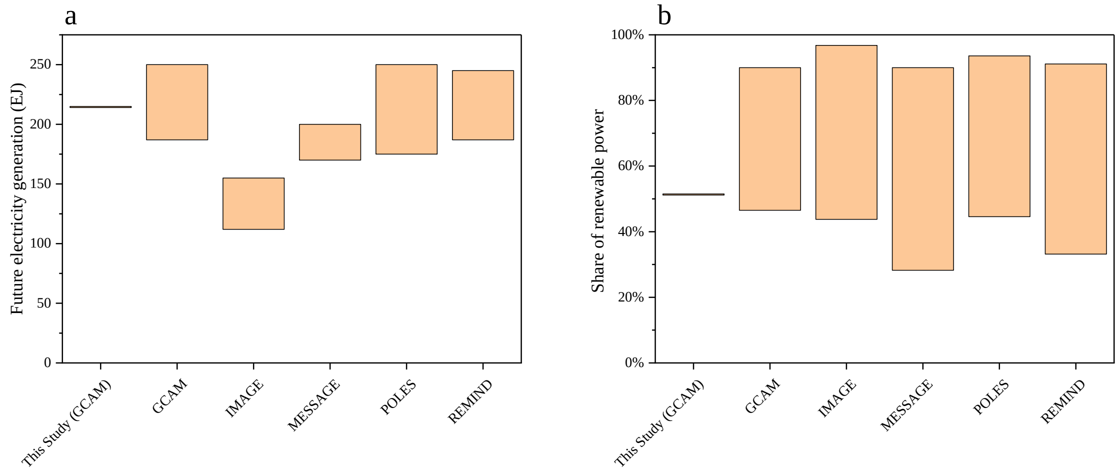

Supplementary Figure. 3 Comparison with the results of previous studies. a future electricity generation and **b** share of renewable power. Results comparison (select SSP2+RCP2.6 as a proxy) on global power generation pathways as modelled by five structurally different Integrated Assessment Modelling systems (GCAM, IMAGE, MESSAGE-GLOBIOM, POLES and REMIND)[1-4].

Supplementary References

1. Rauner, S. et al. (2020). Coal-exit health and environmental damage reductions outweigh economic impacts. *Nat. Clim. Change* **10**, 308-312 (2020).
2. Luderer, G. et al. Environmental co-benefits and adverse side-effects of alternative power sector decarbonization strategies. *Nat. Commun.* **10**, 5229 (2019).
3. Cui, R. Y. et al. Quantifying operational lifetimes for coal power plants under the Paris goals. *Nat. Commun.* **10**, 4759 (2019).
4. Tong, D. et al. Health co-benefits of climate change mitigation depend on strategic power plant retirements and pollution controls. *Nat. Clim. Change* **11**, 1077-1083 (2021).
5. World Bank Open Data. Published 2023. Accessed May 08, 2023. https://data.worldbank.org/.
6. United Nations Statistics Division. Global indicator framework for the Sustainable Development Goals and targets of the 2030 Agenda for Sustainable Development. https://unstats.un.org/sdgs/indicators/indicators-list (2021).
7. Sustainable Development Solutions Network. Sustainable Development Report 2020. https://www.sdgindex.org/reports/sustainable-development-report-2020/ (2020).
8. Sustainable Development Solutions Network. Indicators and a Monitoring Framework for Sustainable Development Goals: Launching a data revolution for the SDGs. https://resources.unsdsn.org/indicators-and-a-monitoring-framework-for-sustainable-development-goals-launching-a-data-revolution-for-the-sdgs (2015).
9. Xu, Z. et al. Assessing progress towards sustainable development over space and time. *Nature* **577**, 74-78 (2020).
