# Supplementary material for: The global power sector’s low-carbon transition may enhance sustainable development goal achievement
Source: Nat Commun. 2023 May 30;14:3144. doi: 10.1038/s41467-023-38987-4 (PMC10229651; doi:10.1038/s41467-023-38987-4)
Supplement: Supplementary file 2 — Description of Additional Supplementary Files [file 41467_2023_38987_MOESM2_ESM.pdf]

Description of Additional Supplementary Files

File Name: Supplementary Data 1
Description: The ratio of CO_2 emissions per unit of thermal power generation in 2030, 2050 and 2100 under nine different scenarios to those in 2015

File Name: Supplementary Data 2
Description: The ratio of NO_X emissions per unit of thermal power generation in 2030, 2050 and 2100 under nine different scenarios to those in 2015

File Name: Supplementary Data 3
Description: The ratio of SO_X emissions per unit of thermal power generation in 2030, 2050 and 2100 under nine different scenarios to those in 2015

File Name: Supplementary Data 4
Description: The ratio of PM emissions per unit of thermal power generation in 2030, 2050 and 2100 under nine different scenarios to those in 2015

File Name: Supplementary Data 5
Description: The ratio of blue water withdrawal per unit of thermal and nuclear power generation in 2030, 2050 and 2100 under nine different scenarios to those in 2015

File Name: Supplementary Data 6
Description: The ratio of blue water consumption per unit of thermal and nuclear power generation in 2030, 2050 and 2100 under nine different scenarios to those in 2015

File Name: Supplementary Data 7
Description: The ratio of power generation in 2030, 2050 and 2100 under nine different scenarios to those in 2015
